# Phosphatase and tensin homologue: a therapeutic target for SMA

**DOI:** 10.1038/sigtrans.2017.38

**Published:** 2017-09-08

**Authors:** Vinay K Godena, Ke Ning

**Affiliations:** 1 Department of Neuroscience, Sheffield Institute of Translational Neuroscience (SITraN), University of Sheffield, Sheffield, UK

## Abstract

Spinal muscular atrophy (SMA) is one of the most common juvenile neurodegenerative diseases, which can be associated with child mortality. SMA is caused by a mutation of ubiquitously expressed gene, *Survival Motor Neuron1* (*SMN1*), leading to reduced SMN protein and the motor neuron death. The disease is incurable and the only therapeutic strategy to follow is to improve the expression of SMN protein levels in motor neurons. Significant numbers of motor neurons in SMA mice and SMA cultures are caspase positive with condensed nuclei, suggesting that these cells are prone to a process of cell death called apoptosis. Searching for other potential molecules or signaling pathways that are neuroprotective for central nervous system (CNS) insults is essential for widening the scope of developmental medicine. PTEN, a Phosphatase and Tensin homologue, is a tumor suppressor, which is widely expressed in CNS. PTEN depletion activates anti-apoptotic factors and it is evident that the pathway plays an important protective role in many neurodegenerative disorders. It functions as a negative regulator of PIP3/AKT pathway and thereby modulates its downstream cellular functions through lipid phosphatase activity. Moreover, previous reports from our group demonstrated that, PTEN depletion using viral vector delivery system in SMN delta7 mice reduces disease pathology, with significant rescue on survival rate and the body weight of the SMA mice. Thus knockdown/depletion/mutation of PTEN and manipulation of PTEN medicated Akt/PKB signaling pathway may represent an important therapeutic strategy to promote motor neuron survival in SMA.

## Introduction

Spinal muscular atrophy (SMA) is a neuromuscular disorder caused by reduced Survival motor neuron (SMN) protein levels. It is an autosomal recessive pediatric neurological disorder characterized by motor neuron degeneration, muscle wasting and paralysis that can progress very rapidly to early childhood death.^1–3^ SMA is classified into four types, based on the manifested clinical symptoms and the age of onset.^4^ Type-1 is the most common form characterized by severe, generalized muscular weakness and hypotonia. It can cause problems in movement, eating, breathing and inability to sit without support. Death from respiratory failure usually occurs within first few years. Type-2 usually appears in an infant with the age of about 7–18 months. Children affected by type-2 SMA are usually able to sit, but cannot stand or walk without a proper support. They have breathing difficulties and shorter life expectancy. Type-3 is a milder form of the disease, and the symptoms may not appear until 18 months of age. Children with type-3 SMA can stand and walk unaided but developing muscle weakness eventually leads to inability to stand or walk. This type of the disease does not usually affect the life expectancy. Type-4 is adult onset and is less frequent than the other types. The symptoms including muscle weakness and movement difficulties are very mild and life expectancy is unaffected.

SMN is an essential cellular protein. The 38 kDa SMN protein is expressed both in cytoplasm and the nucleus of many cell types and is involved in many aspects of RNA metabolism.^5^ The protein is a part of SMN complex with gemin proteins and plays an important role in small nuclear ribonucleoprotein (SnRNP) biogenesis and pre-mRNA splicing,^6–8^ it is still unclear why in SMA the SMN deficiency specifically affects motor neurons. Though the specific vulnerability of motor neurons is still unknown, it has been suggested that SMN requirement is higher in motor neurons than the other tissues due to its function in splicing of the transcripts required specially for motor neuron differentiation, neural development and axonal transport.^9,10^ The interaction of SMN with hnRNPs delivers β-actin to presynaptic nerve terminals, which is essential for axonal elongation and determining the size of growth cone. The amount of β-actin mRNA and protein at axonal terminals and growth cones is significantly reduced in severe SMA mice, suggesting the role of SMN in transport and local translation of axonal mRNA at synaptic terminals.^11^

## SMN splicing and splice variants

There are two identical copies of SMN genes, SMN1 and SMN2 in humans.^12^ But homozygous deletion, rearrangement or mutation of SMN1 is the major cause of the disease in patients.^12^ Though SMN1 and SMN2 have the capacity to encode a functional protein, only SMN1 acts as a protein-determining gene. A silent non-polymorphic nucleotide difference (C to T substitution) at position six of Exon 7 (Ex7+6) in SMN2 disturbs splice enhancer in > 90% of SMN2 transcripts, which ultimately leads to alternative splicing and truncated unstable protein lacking exon 7 called SMN delta7. The exonic splicing enhancer (ESE) in SMN1 is recognized by positive splicing factor 2 (SF2) or alternative splicing factor (ASF). In SMN1, SF2/ASF interaction with U2 class of small ribonuclear protein (U2 snRNP) under the influence of positive splicing factors such as SR rich proteins and Tra2, maintain the Exon7 inclusion. Whereas in SMN2 the C to T substitution favors binding of a negative splicing factor known as heterogeneous nuclear ribonuclear protein A1 (hnRNP A1). So SF2/ASF no longer recognize the enhancer sequence and fail to interact with other positive splicing factors resulting a SMN2 mRNA transcript without Exon7.^13^ This biochemically defective splicing event of SMN2 produces considerably less protein (<10%), which is not sufficient to sustain the survival and function of motor neurons. Thus, in the absence of SMN1 the severity of disease pathology mainly depends on SMN2 copy number.^14^ Keeping this in mind, a number of strategies have been developed recently to induce Exon7 inclusion in SMN2. One of these is the introduction of bifunctional RNAs to increase the SMN splicing efficiency. During SMN2 splicing, binding of hnRNP-A1 to the 5′ end of Exon7 decreases the splicing efficiency at acceptor site. Bifuctional RNAs, in which one domain carries an antisense RNA sequence specific to a target RNA (hnRNP-A1) and the other domain provide binding platform for splicing factors (SF2/ASF, hTra2beta1), increase the splicing efficiency. Using *in vivo* delivery system, plasmid based recombinant Adeno-associated virus vectors (rAAV) have been designed to express bifunctional RNAs to stimulate SMN2 Exon7 inclusion and functional SMN protein expression in patient fibroblasts.^15^ In contrast to humans, mice and other mammalian species have a single SMN gene, which is equivalent to human SMN1.^16,17^ Homozygous loss of this gene in mice is embryonic lethal, indicating the necessity of SMN gene product for cell survival and function. A mouse model suitable for human SMA was developed by introducing a transgenic human SMN2 into SMN null background.^18^ At birth, these animals have normal neuromuscular system but with increasing age they develop delayed NMJ maturation with neurofilament (NF) aggregates, reduced neurotransmitter release, failed acetylcholine receptor (AChR) clustering and die with severe muscle paralysis within a week or 10days of age.^19,20^ Introduction of two or more copies of SMN2 in knockout background rescue the embryonic lethality and survival respectively, suggesting the importance of SMN functional role and also the significance of the copy number.

## Neuronal cell death/apoptosis in SMA

SMA model systems and SMA cultures show a significant decline in motor neurons with increasing age and between 3 and 10 weeks of differentiation, respectively.^21–23^ The reason could be either the developmental maturation error or death of motor neurons over time. It is evident that greater number of SMA iPSC differentiated motor neurons were caspase positive with a significant chromatin condensation compared to the controls suggesting active apoptosis during motor neuron development or maturation.^22,24^ Moreover, the neuronal apoptosis inhibitor protein (NAIP) on chromosome 5 was mutated in a greater number of SMA type 1 cases,^25^ suggesting a direct link between apoptosis and SMA. Apoptosis is a complex process of programmed cell death, mediated by the activation of a family of proteases known as caspases. The apoptotic cascades can be triggered upon ligand binding such as TNF or FasL to their death receptors TNFR or Fas, respectively. In mitochondria this cascade is induced by intracellular stresses and by various proteins such as Bcl-2, Bcl-c, Bax and the tumor suppressor p53. Using a murine embryonic carcinoma cell line P19, a direct effect of SMN loss on apoptotic cell death was observed.^24^ In SMN knocked down differentiated P19 cell neurons the cell death was noticed as caspase dependent, which is confirmed by using pan-caspase inhibitor ZVAD-fmk, which led to a significant reduction in the rate of apoptosis in SMN knocked down cells. In addition, the same neurons also displayed intrinsic mitochondrial mediated apoptosis.^24^ Cytochrome-c is a member of mitochondrial electron transport chain and also a mediator of caspase cascade. Cytochorme-c staining in differentiated P19 cell neurons displayed patchy and clumped cytochrome *c* accumulation in cytoplasm with condensed nucleus, while the control cells showed a punctate distribution of cytochrome *c* with large smooth nuclei, indicating an intrinsic mitochondrial apoptosis in RNAi treated cells. Similar to this, in another study where the author used iPSCells generated from a couple of type-1 SMA patients. One produced with lentiviral constructs and the other using a virus-free plasmid based approach.^22^ Both the iPSC lines have significantly fewer motor neurons at later stages of differentiation compared to control iPSC-derived motor neurons. The cultures showed an increase in the activation of Fas ligand mediated apoptotic pathway through caspase-8 and caspase-3. Blocking this pathway by using anti-Fas monoclonal antibody (Fas NT Ab) and caspase-3 specific inhibitor Z-DVED-FMK significantly increase the motor neuron number,^22^ suggesting that apoptosis plays an important role in SMA pathology and therapies targeting this cascade may have significant clinical applications.

## SMA disease pathology and therapeutic strategies

Though initially it was considered as a specific motor neuron disease recent studies revealed that the disease is not specific to motor neurons rather is a multi organ disorder.^26,27^ This is due to the involvement of other neuronal population and vital organ systems in SMA disease pathology. Various studies provided evidences that the neurodegenerative disorder in SMA is also due to loss of sensory neurons, inter neurons and disturbed astrocytic function.^28,29^ Moreover, there was an evidential claim that the activation of morphological and cellular alteration of astrocytes in SMN delta7 mouse and SMA-induced pluripotent stem cells are prior to motor neuron loss.^29^ Viral vector based restoration of SMN protein in astrocytes increases the survival and neuromuscular connections in SMA models indicating a direct contribution of astrocytes to the disease pathogenesis.^30^ In SMA, the disease pathogenesis also includes a severe muscle phenotype such as a significant delay in muscle growth, muscle development and maturation. In addition to this the muscle fibres are defective and disorganized.^31^ This may be either a direct SMN deficiency effect on muscle or an indirect effect through motor neuronal dysfunction that contributes muscle atrophy. Cardiac system is an important vital organ system to consider in SMA pathology. This is due to the appearance of organ structural defects well in advance of motor neuron dysfunction. Constant appearance of congenital septal defects, arrhythmia and cardiomyopathy are the most important cardiac phenotypes noticed in patients with severe SMA and SMA mouse models.^32,33^ Adding to this vascular defect such as distal digital necrosis, where the necrotic lesions occur at the distal aspects of fingers or toes are also commonly reported in severe SMA patients.^34^ Viral delivery systems carrying SMN (scAAV9-SMN) at the early post-natal stage prevented these cardiac severe defects and significantly improved the survival, suggesting the role of SMN in cardiovascular system. Other organs involved in SMA pathology are liver and pancreas. Abnormal fatty acid metabolism, acute pancreatitis, diabetes and alterations in glucose metabolism are some of the important pathological defects noticed in SMA.^35–37^ Further, the story of SMA disease pathology extended to bone as well. High incidence of fractures, hypercalcemia and severe osteopenia are the important symptoms noticed in patients affected by SMA.^38,39^ Based on these evidences it is clear that in addition to motor neurons, restoration of SMN is required in many other cell and tissue types. Further investigation is needed to improve the disease modifying treatment for SMA.

**Figure 1:**
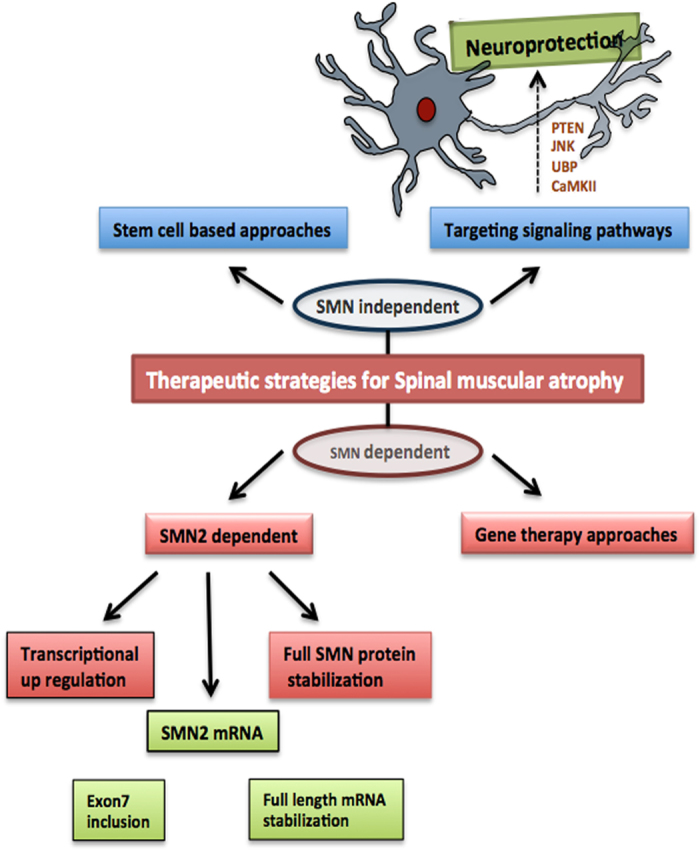
Possible therapeutic strategies for SMA. The SMN dependent strategies concentrating mainly on SMN2 gene expression, whereas the SMN independent treatment is concentrating on different possible signaling pathways that are capable to modulate the disease pathology in SMA.

SMA is currently incurable. This is because of the severity of effects on the neuromuscular system and other organ systems and lack of methods for efficient delivery of therapeutically functional molecules. Most of the drugs are still under clinical trials and as such there is no effective treatment of the disease so far. So there is an essential requirement to identify the therapeutic strategies that delay the advice of SMA pathology (Figure 1). Gene therapy approaches using different viral vectors increases the expression of the SMN and significantly improve the survival in mice.^40,41^ However, in order to get a significant increase in SMN gene expression, reduced disease pathology and improved survival rate of the mice, the viral vectors must be delivered on postnatal day1.^42^ The injections on postnatal day5 and day10 have little or no effect respectively, suggesting that early detection and the immediate therapy before noticing the clinical symptoms is required. For that the early detection program and newborn screenings must be established in order to avoid further consequences.^4^ Though restoring SMN expression rescues early lethality in SMA mouse model, there will be an adequate requirement to find out alternative mechanisms and molecular pathways that provide additional protection to the motor neurons, evade muscle degeneration and also to find potential therapeutic targets (Figure 1).

Recent studies on c-Jun NH2-terminal kinase 3 (JNK3) revealed that this pathway mediates neurodegeneration in SMA. JNK3 is a neuron specific isoform mediates neurodegeneration in SMA mice caused by low SMN levels. JNK3 deficiency reduces the neuronal degeneration and rescues the SMA phenotype without altering levels of SMN. The deficiency also reduces the muscle atrophy, improves muscle fiber thickness, the overall growth and the lifespan of SMA mice.^43^ JNK3 represents a potential therapeutic target for SMA treatment. The other important pathway involved in SMA pathology is Ubiquitin signaling cascade. Dysregulated ubiquitin homeostasis represents a key driver of both neuromuscular and systemic pathology in SMA.^21,44^ Ubiquitin activating enzyme 1 (UBA1) is a part of ubiquitin protein degradation pathway and also an important protein involved in neuronal cell growth and differentiation. UBA1 suppression in iPSC derived human SMA motor neurons, non-SMN dependent form of SMA in human patients with UPA/UBA1 mutations^45^ and accumulation of UPS target protein in SMA patients due to dysregulation of UPS/UBA1 pathway^46^ suggesting UBA1 as an important and potentially attractive therapeutic target for SMA. In addition to reduced levels of UBA1 in SMA motor neurons, the cellular distribution of UBA1 is also altered. UBA1 is localized mainly in the cytoplasm in contrast to the nuclear distribution in control motor neurons. Nuclear accumulation of UBA1 mainly correlates with neuronal maturation and differentiation of motor neurons.^21^ In SMA motor neurons due to cytoplasmic distribution of UBA1 there is a developmental delay or abnormality in these cells. Ubiquitin Carboxyl-Terminal Esterase L1 (UCHL1) is the other protein of UPS complex involved in SMA pathology and it is also noticed as reduced in SMN motor neurons derived from patient fibroblasts. UCHL1 is involved in cell machinery to degrade unwanted proteins and it has two types of enzymatic activity. The hydrolase activity removes and recycles the ubiquitin molecules from the degraded proteins whereas; the ligase activity links the activity of ubiquitin molecules for use in tagging proteins for disposal. Patients with loss of UCHL1 demonstrated early onset of neurodegeneration^47^ and exacerbate disease symptoms in mouse model of SMA. Preliminary studies on zebrafish demonstrated UBA1 restoration rescue of the functional and morphological neuromuscular defects of SMA fish.^44^ AAV9 mediated delivery of UBA1 in mice restores the gene levels, rescues the neuromuscular defects, improves the body weight, survival and motor performance. The AAV9-UBA1 treated SMA mice also improved the systemic pathology in heart and liver. Moreover, the study demonstrates the modest increase in the expression of full-length SMN mRNA and SMN protein up on UBA1 delivery in SMA mice.^44^ Similar to JNK3 pathway and Ubiquitin signaling cascade, here we proposed the PTEN mediated Akt/PKB signaling pathway which plays an important role in axonal growth and stability of motor neurons through the regulation of its downstream cellular functions.

## PTEN structure and signaling pathway

**Figure 2:**
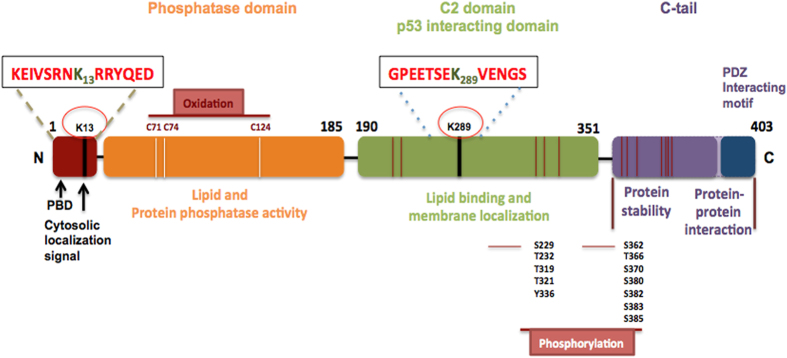
PTEN structure. A structure of 403 amino acids with N-terminal, Phosphatase, C2 domains and the C-terminal tail. The N-terminal domain contains PIP2 binding domain (PBD) and an important ubiquitination site K13 responsible for nuclear translocation. The Phosphatase domain of PTEN is responsible for its protein and lipid phosphatase activities. The C2 domain is hosting p53-binding site, which is responsible for PTEN apoptotic activity. Other important sites labeled with magenta are the phosphorylation sites both in C2 domain and the C-ter tail. These residues are phosphorylated by Glycogen synthase kinase 3B (GSK3b) and casein kinase 2 (CK2). C-ter tail of PTEN is responsible for protein stability and protein–protein interactions.

PTEN (a phosphatase and tensin homologue deleted on chromosome 10), is a tumor suppressor with both lipid and protein phosphatase activities. PTEN structure consists of a Phosphatase domain and a C2 domain responsible for its enzymatic function and phospholipid membrane binding respectively (Figure 2). The amino terminal end (N-ter) defines the catalytic activity of the protein, whereas the carboxy terminal (C-ter) with PDZ domain deals with protein-protein interactions.^48^

**Figure 3:**
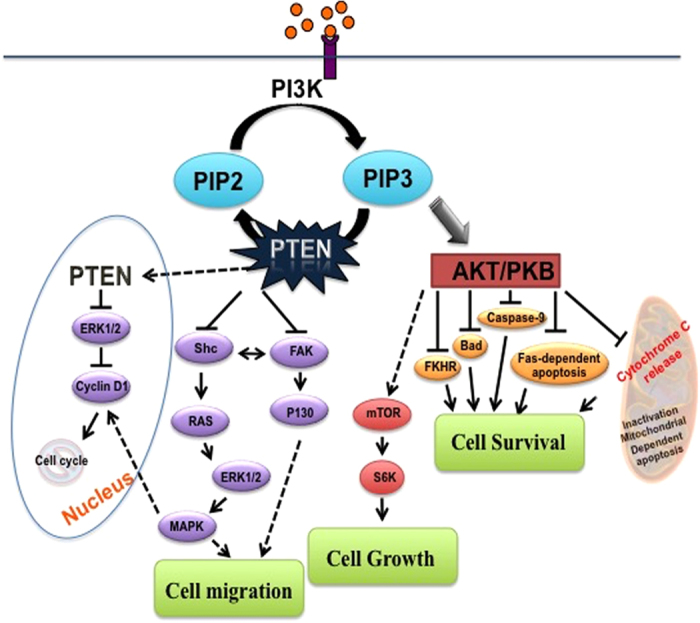
Schematic presentation of suggested pathways of PTEN involvement in neuronal survival. PTEN regulates the conversion of Phosphatidylinositol (3,4,5) triphosphate into Phosphatidylinositol (4,5) bis phosphate (PIP2). PTEN activation leads to cell spreading and growth by inhibition or dephosphorylation of FAK1 (protein tyrosine kinase 2) and SHC transforming protein 1 in cytoplasm and by inhibiting cyclin D1 in the nucleus. PTEN depletion activate Akt signaling pathway which in turn regulate all downstream cellular effects such as cell growth, proliferation and cell survival. PTEN depletion is sufficient to trigger mammalian target of rapamycin (mTOR) that alters downstream signaling pathways for local protein synthesis by increasing P70s6 kinase activity. This pathway also influences many apoptosis factors either by transcriptional regulation or by direct phosphorylation.

PTEN is highly expressed in the neurons of adult CNS. It is localized both in the nucleus and the cytoplasm of neuronal and glial cells.^49^ The expression of PTEN is ubiquitous and it is required for cell proliferation, growth and migration from early embryonic developmental stages. PTEN functions as a phosphatase to dephosphorylate the phosphatidylinositol (3,4,5) tri phosphate (PIP3) to phosphatidylinositol (4,5) bis-phosphate (PIP2).^50^ PIP3 is an important primary activator of Akt/PKB, a serine/threonine kinase pathway (Figure 3). PTEN inhibit Akt signal transduction pathway and modulates its downstream cellular functions through lipid phosphatase activity. Cell motility via small G proteins RAC1 and CDC42, also depending on PTEN lipid phosphatase activity.^51^ The downstream effect of increased PIP3 by PTEN inhibition activates many pathways, which include mTOR mediated cellular signaling;^52^ controlled apoptosis pathway either by transcriptional regulation or by phosphorylation;^53^ neuronal polarity through GSK3 inhibition;^54^ proliferation and self-renewal of various cell and progenitor cell types;^55^ laminin and cytoskeletal protein expression;^56^ glycolysis and glycogeneis;^57^ TGF-β and BMP associated SMAD pathways.^58^ The protein phosphatase activity of PTEN includes, dephosphorylation of focal adhesion kinase (FAK) in cytosol, Shc dephosphorylation, and the MAP kinase activity through RAS/ERK pathway in the nucleus^59^ (Figure 3).

## PTEN neuroprotection in neurological disorders

Studies on gliomas and medulloblastomas raise the importance of PTEN in developmental biology. Further, the germ line mutations of this protein in brain disorders demonstrate the significance of PTEN in CNS and established a proper study model for neurodegenerative diseases. PTEN mutations display brain disorders such as macrocephaly, neuronal hypertrophy, seizures, abnormal social interactions and mental retardation. On the other hand recent reports clearly evidenced the PTEN-down-regulation mediated neuro protection using both *in vitro* and *in vivo* model systems. Briefly, in Lou Gehrig’s disease popularly known as amyotrophic lateral sclerosis (ALS), excitotoxicity is considered to play a key role where, AMPA receptors allow cytotoxic levels of calcium into motor neurons and contributes neuronal injury.^60^ GluR2, one of the heteromeric receptor complexes of AMPA determines calcium permeability and selective death of MNs in ALS. PTEN knock down in primary cultured motor neurons and induced pluripotent stem cell (iPSC)-derived motor neurons attenuates the death of these neurons by decreasing the translocation of GluR2 receptor subunit into the membrane. This study of induced pluripotent stem cells (iPSc) differentiated motor neurons open gates to new approaches in ALS therapy.^61^ In age-related neurodegenerative disorder like Parkinson’s disease, oxidative stress due to PTEN accumulation in mitochondria associated with Bax, cytochrome *c* and activated Caspase 3 leads to mitophagy, which is the primary reason for neurodegeneration in patients. Knockdown of PTEN inhibits Caspase 3 activation through Akt signal transduction pathway and prevents neuronal damage.^53^ Also, selective PTEN depletion in dopaminergic neurons in mouse model of Parkinson’s disease exhibit more extensive pattern of neurite outgrowth and are less susceptible to cell death. The selective deletion of PTEN in dopamine neurons enhances the survival, neurite outgrowth, function and integration of grafted neurons with the host tissue.^62^ This strategy of transplantation of modified neurons in to animals to improve graft viability and function in cell replacement therapies are promising.

In addition to its protective role after neuronal injury, PTEN depletion or inactivation also enhances neurogenesis and neuroregeneration. Failure of injured axons to regenerate in the CNS leads to paralysis, which is the characteristic feature of spinal cord injury. Regeneration usually takes place either by sprouting spread of non-injured axon to form new circuits or regeneration of lesion axon to reform the lost connections. Delivery of neurotrophic factors, removing extracellular inhibitory molecules^63,64^ and over expressing BDNF receptor TrkB^65^ can promote some degree of regeneration but is limited. In cortical neurons axotomy diminish mTOR activity, which is the central regulator of cap dependent protein translation that leads to defective regenerative ability in adult CNS. PTEN inactivation or deletion activates downstream pathways, such as Akt and mTOR signaling in these neurons and promotes protein synthesis for axonal regeneration.^54,66^

## PTEN downregulation to SMA pathogenesis

**Figure 4:**
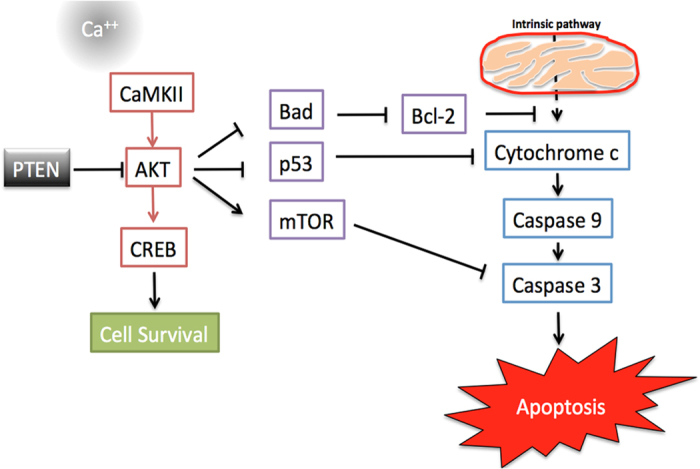
General overview of the process of cell death in SMA. The activation of intrinsic and extrinsic apoptotic pathways up on stimulation. The intrinsic pathway in mitochondria activates cell death via caspase 8 and caspase 3. In extrinsic pathway the activation of caspases is due to different signalling molecules such as increased calcium, reactive oxygen species and excitotoxicity. PTEN depletion activates PIP3/AKT signaling pathway that blocks the caspase mediated apoptosis pathway.

The process of cell death named Apoptosis has been implicated in many neurodegenerative diseases including SMA (Figure 4). Loss of SMN1 leads to motor neuron cell death through apoptosis.^54^ Increased levels of membrane bound FasL, activation of Caspase-8 and Caspase-3 and chromatin condensation in SMA-iPSC differentiated motor neurons provide a direct evidence of apoptosis in SMA pathology.^22^ The lack of apoptosis in PTEN mutated tumors raise the hope of this tumor suppressor gene in regulating neuronal apoptosis during neurodegeneration. Recent literature has shown that this tumor suppressor protein considered to be an important target because of its neuro-protective effect^51^ and also its controlling effect on Akt phosphorylation and its downstream cellular functions. PTEN is a crucial mediator of mitochondria-dependent apoptosis. Studies using Staurosporine (STS) induced apoptotic damage in hippocampal neurons demonstrated that the suppression of PTEN expression significantly reduces the reactive oxygen species (ROS) and interfered with mitochondrial apoptotic cascade by inhibiting cytochrome *c* release and caspase 3 activation.^53^ They provided a clear evidence of direct association of PTEN with Bax, the pro-apoptotic protein of Bcl-2 family members. In addition to Bax, other pro-apoptotic factor p53 has direct association with PTEN.^50^ Adding to that the C2 domain of PTEN contains p53-binding site by which the cross talk taking place during apoptosis.^67^ PTEN inhibition activates the anti-apoptotic factors and plays a neuroprotective role in focal ischemia and global cerebral ischemia.^22^ Gene therapy based approaches of blocking Akt mediated apoptosis pathway through PTEN depletion may become one of the possible therapeutic strategies. Further studies on PTEN regulation through signal transduction mechanism will be important and helpful in developing therapeutic targets for SMA disease pathology and other neuronal disorders.

Other important milestone of PTEN research is the identification of its ubiquitination sites, which are important for PTEN nuclear translocation. During tumorigenesis, the reduced nuclear translocation of PTEN impairs its tumor suppressing function, leading to reduced apoptotic cell death, suggesting that PTEN nuclear translocation is causally linked to its apoptotic activity.^68^ Though PTEN has two important ubiquitination sites K13 and K289 (Figure 2) the C2 domain ubiquitination site PTEN^K289^ neither is involved in nuclear translocation nor in reducing apoptosis. PTEN Mono-ubiquitination of PTEN at lysine-13 residue (K13) upon excitotoxic stimulation of *N*-methyl-D-aspartate (NMDA) is required for PTEN nuclear translocation in cultured neurons. It was hypothesised that upon excitotoxic/ischemic brain insult, PTEN may translocate to nucleus and its accumulation may cause neuronal damage. Keeping this in mind Wang YT and colleagues designed a short interfering peptide that flanks K13 residue (tat-K13), which may competitively inhibit PTEN mono ubiquitination and hence block its nuclear translocation.^69^ PTEN tat-K13 peptide reduces the NMDAR-mediated excitotoxicity and blocks PTEN nuclear translocation in cultured neurons and protects against ischemic brain injury in rats. The concept of preventing apoptosis or neuronal death by blocking PTEN nuclear translocation is a new concept and it may have broad implications in other neurodegenerative disorders including SMA.

Activation of Akt upon PTEN depletion triggers mTOR pathway, which is sufficient to raise β-actin and axonal growth in MNs. This approach of PTEN depletion using viral vector in SMNdelta7 mice achieved a substantial improvement of motor neuron survival and represent an important therapeutic strategy to promote the health of motor neurons in SMA and other motor neuron diseases.^70,71^ Branchu *et al.*^72^, in their study demonstrated that the drug inhibition of MEK/ERK/Elk-1 signaling pathway could be beneficial to study the severe types of SMA. Inhibition of extracellular signal regulated kinase (ERK) pathway promotes the PI3K/AKT/CREB pathway activation and increases the SMN expression in SMA mice spinal cord and human SMA myotubes. The activation of PI3K/AKT pathway also increases the lifespan of SMA mice. MEK/ERK/Elk-1 and PI3K/AKT both recruit the cAMP element binding protein (CREB), which is considered to be a powerful activator of neuron pro survival transcription factor and an efficient trans activator of the SMN gene.^73^ This shift of ERK to AKT is mediated by additional transcription factor CaMKII, which is directly involved in AKT activation after ERK inhibition. CaMKII mediated activation of AKT/CREB pathway is acting parallel to the PTEN mediated PIP3/AKT pathway, which is considered to be one of the targeting cascade of therapeutic applications. Interestingly PTEN knockdown using scAAV9-siPTEN improved the motor function of SMA mice and significantly increase the lifespan in SMA mice.^70,71^ Finally it is tempting to speculate that combined delivery of siPTEN along with coSMN may show result in potential improvement in disease pathology and will be helpful pharmacologically in drug development.

## Conclusion

Disease models of SMA suggested that reduced SMN is the main cause of disease pathology. Different model systems with a variety of strategies improved the quality of selective spinal motor neurons and also extended the lifespan *in vivo.* Therapeutic approaches concentrating to raise the levels of SMN protein and also for the betterment in screening methods in newborn. Parallel to this other studies concentrating on intrinsic signaling pathways to target the motor neuron quality control via regulation of protein synthesis. Tumor suppressor protein PTEN is a negative regulator of Akt phosporylation, is one of the promising therapeutic target in developmental medicine. PTEN depletion enhances the axonal growth and improves survival in SMN-deficient motor neurons. PTEN neuroprotective signaling presents the possibility as a therapeutic target for SMA and other neurodegenerative diseases. Next-generation therapeutic strategies such as using combinatorial drug treatment may helpful in drug discovery. And also further studies using a model system, which is close to human disease model such as iPSCs will be helpful to understand the disease pathology, molecular mechanism, drug discovery, toxicology studies and therapeutic use.
